# From the Laboratory to the Field: Assessing the Usability of the NTDscope in the Tropical Rainforest Region of Gabon

**DOI:** 10.4269/ajtmh.25-0596

**Published:** 2026-06-23

**Authors:** Rella Zoleko Manego, Lillian Rene Endamne, Victor Pahl, María Díaz de León Derby, Ghyslain Mombo-Ngoma, Daniel A. Fletcher, Isaac I. Bogoch, Michael Ramharter, Saskia Dede Davi

**Affiliations:** ^1^ Centre de Recherches Médicales de Lambaréné, Lambaréné, Gabon;; ^2^ Center for Tropical Medicine, Bernhard-Nocht Institute for Tropical Medicine & I. Department of Medicine, University Medical Centre Hamburg–Eppendorf, Hamburg, Germany;; ^3^ German Centre for Infection Research, Partner Site Hamburg–Lübeck–Borstel–Riems, Hamburg, Germany;; ^4^ Department of Bioengineering, University of California, Berkeley, California, USA;; ^5^ Drug Implementation Research Group, Bernhard-Nocht Institute for Tropical Medicine & I. Department of Medicine, University Medical Centre Hamburg–Eppendorf, Hamburg, Germany;; ^6^ Biological Systems and Engineering Division, Lawrence Berkeley National Laboratory, University of California, Berkeley, California, USA;; ^7^ Chan Zuckerberg Biohub, San Francisco, California, USA;; ^8^ California Institute for Quantitative Biosciences, University of California, Berkeley, California, USA;; ^9^ Division of Biological Systems and Engineering, Lawrence Berkeley National Laboratory, Berkeley, California, USA;; ^10^ Division of General Internal Medicine, Toronto General Hospital, University Health Network, Toronto, Canada;; ^11^ Division of Infectious Diseases, Toronto General Hospital, University Health Network, Toronto, Canada;; ^12^ Department of Medicine, University of Toronto, Toronto, Canada

## Abstract

In regions where *Loa loa* is coendemic with lymphatic filariasis and onchocerciasis, mass drug administration of ivermectin has posed a risk for serious adverse reactions in individuals with high microfilarial loads. To overcome this problem, easy-to-use point-of-care diagnostic tools are crucial for identifying individuals at risk in population-based control programs. The NTDscope is a device that uses smartphone components for video microscopy and may support decentralized *L. loa* microfilariae screening under field conditions. This study evaluated the usability and operational feasibility of the NTDscope during population-based screening in a rural setting. A total of 200 adults were screened and 187 adults were enrolled locally by six trained health care workers with no experience using the device. From blood collection to automated analysis, the process was assessed to evaluate usability and identify potential challenges in field deployment. The device was well accepted by health care workers and participants because of its intuitive interface, portability, and rapid result output. Operational challenges included environmental factors and minor technical limitations. Nonetheless, the device performed reliably in the field without reliance on internet connectivity or electricity and required minimal training. Feedback highlighted the need for improved heat management, optimized error recognition, and enhanced data security. The NTDscope proved highly usable in low-resource settings where infection with *L. loa* is most endemic and offers significant potential for supporting large-scale treatment programs in *L. loa*-coendemic regions. Although this study focused on field usability, future research should evaluate the implementation in the frame of large-scale treatment.

## INTRODUCTION

Loiasis is a chronic vector-borne infection caused by the filarial nematode *Loa loa*, which is transmitted by deerflies (*Chrysops* spp.) that bite preferentially during hot daytime hours.^1^ *Loa loa* infection occurs in regions of West and Central Africa, placing 42 million people residing in high-or intermediate-transmission regions at risk of infection. The countries most affected by infection with *L. loa* are Gabon, Equatorial Guinea, Cameroon, the Central African Republic, the Democratic Republic of the Congo, and the Republic of the Congo.^2^^,^^3^ *Loa loa* infection can be classified as microfilaremic defined by the presence of microfilariae circulating in the peripheral blood or as occult defined by the absence of microfilariae in peripheral blood. The clinical standard to determine an infection with *L. loa* is the microscopic assessment of thick smears by trained microscopists.^4^^,^^5^ For many years, infection with *L. loa* has been seen as a benign disease and has received little attention.^6^^,^^7^ Infection with *L. loa* attracted attention during mass drug administration (MDA) campaigns in the 1990s aimed at eliminating lymphatic filariasis and onchocerciasis.^8^^–^^12^ These MDA programs administered ivermectin to large numbers of residents in coendemic regions.^8^^,^^12^ Despite ivermectin’s general safety profile, its use in MDA programs has been linked to serious neurological adverse events in *L. loa-*coendemic areas.^11^^–^^13^ Since the early 1990s, reports have identified more than 500 individuals experiencing a characteristic form of *L. loa* encephalopathy after ivermectin administration, with approximately 60 of these cases proving to be fatal.^13^ These neurological complications have preferentially occurred in individuals with *L. loa* microfilarial counts exceeding 30,000 microfilariae per milliliter (mf/mL) of blood.^13^ The underlying mechanisms are thought to involve eosinophil-driven inflammation around dying parasites, microvascular blockage, and resulting vascular damage within the central nervous system.^2^ According to current WHO recommendations, ivermectin-based MDA is permitted in regions with meso- or hyperendemic onchocerciasis as the anticipated public health gains are considered to outweigh the risk of adverse events.^13^ Nonetheless, the guidelines mandate increased vigilance for detecting and managing such events.^13^ However, vast regions across Central Africa are highly endemic for *L. loa* infection, whereas the endemicity of onchocerciasis is low.^4^^,^^13^ Gabon is a typical example, where infection with *L. loa* prevalence may reach 80% of the adult population in high-risk communities residing in the tropical rainforest region.^4^^,^^5^ However, in Gabon, the overall prevalence of onchocerciasis is confined to only a few villages with active transmission.^14^ The implementation of MDA in such regions thus poses a significant challenge.^13^

Kamgno et al.^13^ have suggested a “test-and-not-treat” approach to address this relevant issue. Using this strategy, individuals with high *L. loa* microfilarial loads were identified and excluded from treatment with ivermectin for the control of onchocerciasis, whereas the majority of the population received treatment with ivermectin safely.^13^ To perform such large-scale treatment requires the preparation of thick smears and assessment with light microscopy by trained microscopists. Implementation of such screening proves challenging in the field or requires significant extra resources if performed in a dedicated laboratory. To overcome this logistical burden, a fast, accurate, point-of-care diagnostic tool is needed that can reliably quantify *L. loa* microfilariae and be easily used by field-workers in population-based screening programs after a brief training. In response to this need, the LoaScope was developed as a first-generation mobile phone-based digital microscope capable of identifying high *L. loa* microfilarial loads within minutes of a capillary blood draw.^15^ The LoaScope was successfully tested in endemic regions of Cameroon.^13^ To expand access to the technology, a next-generation device, which is now known as the NTDscope, was developed with improvements to hardware and software.^16^ This new device is not limited to the diagnosis of *L. loa*. It is designed to expand diagnostic screening to other tropical parasitic infections, such as schistosomiasis, soil-transmitted helminthiasis, and other filaria species. To date, the NTDscope has been technically developed, and the first production batch has been assembled. The device builds on the LoaScope’s mobile phone-based microscopy by incorporating a system-on-module platform that enhances field usability and enables multimodal imaging. This allows for automated, point-of-care detection of *L. loa* directly from patient samples. The overall aim of the NTDscope initiative was to assess *L. loa* microfilarial counts in individuals participating in large-scale treatment and mapping programs to identify those at risk of ivermectin-related serious adverse events (>20,000 mf/mL). This would help to exclude patients at risk of encephalopathy from ivermectin treatment, providing accurate epidemiological information and allowing health care workers to manage *L. loa* infection. Whereas the manuscript published by Díaz de León Derby et al.^16^ focuses on the technology of the device, including sensitivity and specificity assessments of samples gained in this study, this manuscript addresses the usability and practical field implementation of the *L. loa* screening function of the NTDscope under field conditions in Gabon.

## MATERIALS AND METHODS

### The device.

**Figure 1.**
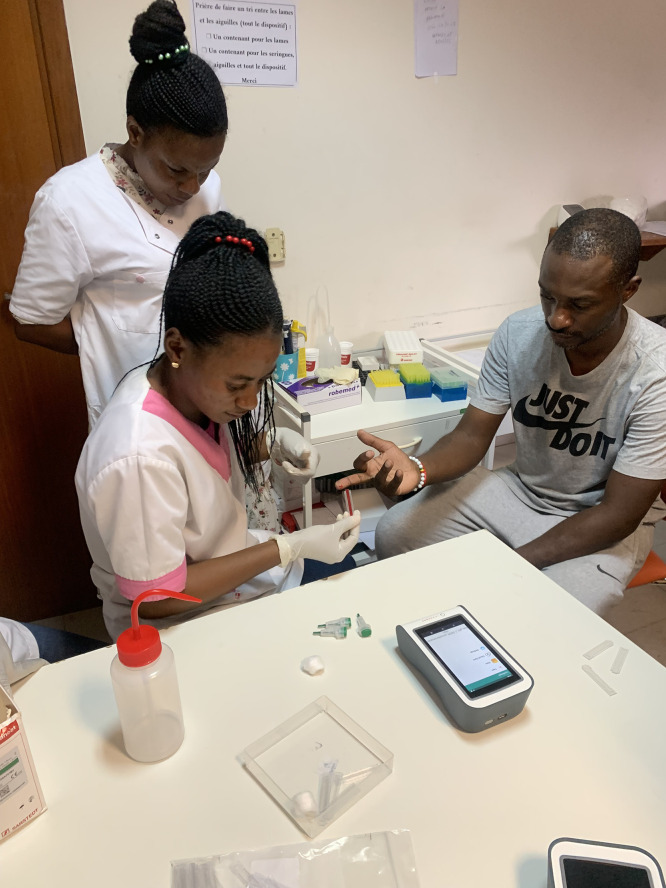
Collection of blood samples for the NTDscope.

The NTDscope was assembled using commercial mobile phone hardware and custom-designed components to enable visual detection of *L. loa* microfilariae in whole blood by automatically capturing and analyzing videos of microfilarial motion. Motorized sample scanning and onboard motion detection using the video function enabled the identification and quantification of microfilariae. Blood was obtained by a finger prick and loaded directly into a small plastic capillary tube without any preparation or staining (Figure 1). Results indicating the presence or absence of high *L. loa* microfilarial counts were available within 2 to 3 minutes of sample processing. More details about the NTDscope are given elsewhere.^16^

**Figure 2.**
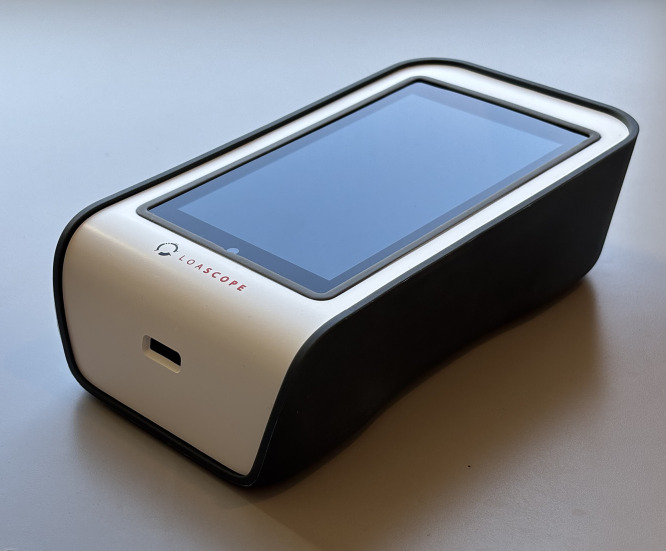
The NTDscope*.* This figure shows the current prototype of the NTDscope.

The NTDscope is easily transportable, weighs 880 grams, and offers portable imaging with multiple contrast modes, allowing for the implementation of digital microscopy (Figure 2). Compared with the LoaScope, the new device does not rely on an entire mobile phone device but rather, integrates selected components, including camera modules, sensors, a rechargeable battery, and a reversed lens system with a broad field of view. The device captures images and videos of samples collected in capillary tubes inserted into the device. The user interface runs as an application on the Android platform, enabling cloud-based data transmission and remote updates. Enhanced processing power allows the implementation of machine learning for automated pathogen detection.

### Training.

**Figure 3.**
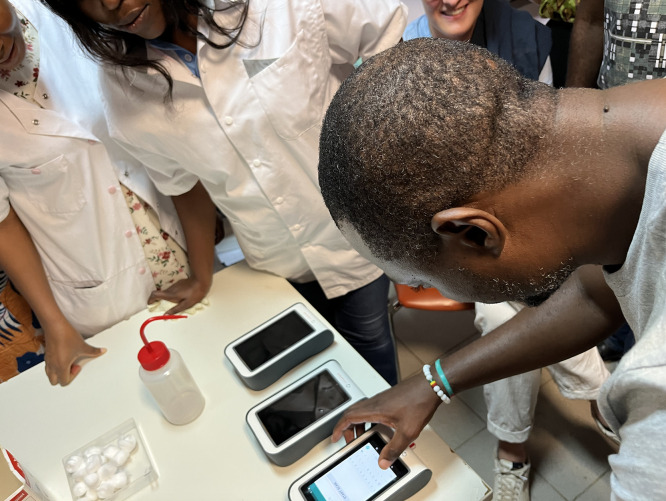
NTDscope training session. A training session on how to use and apply the NTDscope was provided to health care workers who had no prior experience.

For the implementation of a first pilot survey using this new device, a total of six health care workers, including two field-workers, one physician, two laboratory technicians, and one nurse, at the Centre de Recherches Médicales de Lambaréné (CERMEL) were invited for a training program. A field-worker was defined as an individual who conducted research, data collection, intervention delivery, or service provision outside of a controlled laboratory or central office setting and worked directly within the community and natural environment irrespective of education level. The use of the device was first facilitated by a dedicated face-to-face training program that was conducted over 2 days by teams from the University of California, Berkeley and the University of Toronto irrespective of previous knowledge on *L. loa* infections and microscopy. The program included topics on *L. loa* infection, the risks of MDA using ivermectin in *L. loa-*coendemic regions, the relevance of improving rapid screening at the point of care, the technical aspects of the NTDscope, and the practical use of the device, including troubleshooting. Field teams had opportunities to practice using the NTDscope during training in a controlled setting (Figure 3).

### Study sites and target population.

The NTDscope was implemented by CERMEL and its sentinel site, Institut de Recherches en Santé de Sindara, between August 13 and 24, 2023 at the Department of Tsamba Magotsi and Lambaréné, which are both rural areas in Gabon where *L. loa* and *Mansonella perstans* are coendemic. Lambaréné is a small town in Moyen-Ogooué Province.^17^ Sindara is a remote village located in the province of the Ngounié approximately 29 km from Fougamou and 90 km from Lambaréné.^18^ All participants 18 years of age or older were invited to participate after provision of informed consent. For each individual, peripheral blood was obtained using a standard sterile single-use lancet by a finger prick. Two rectangular capillary tubes containing a volume of 43.2 *µ*L were filled with blood and placed into the device for imaging (Figure 1). The device recorded a sequence of videos, which were analyzed using the dedicated NTDscope software. Simultaneously, a separate blood sample was collected to allow for quantification of microfilariae by the routine thick blood smear technique, which served as the reference standard. Participant video samples were evaluated by comparing manual microfilarial counts obtained from NTDscope-acquired videos with microfilariae counts determined from calibrated thick blood smears. A participant was considered microfilariae positive by thick smear if at least one worm of either *L. loa* or *M*. *perstans* was found in the total examined volume. A participant was considered positive on the NTDscope if microfilarial motion was observed at least once in the videos acquired as described elsewhere in detail.^16^

The full screening procedure with the NTDscope, including sample collection, capillary insertion, video capture, and automated processing, was completed in roughly 2 minutes. After the daily screening sessions, the device was charged during the night hours and connected to a Wi-Fi network so that images and videos collected during the screening activities were uploaded to a secured cloud storage. Usability feedback was collected through semistructured group debriefings conducted immediately after training and field implementation. These sessions used an exploratory, open-ended approach to capture authentic user experiences and emerging implementation challenges. These included feedback on explaining the NTDscope to community members at study sites, acquiring samples using capillary tubes, the functionality of capillary tubes within the NTDscope, sample processing time, and overall usability. Specific attention was given to identifying factors that hindered workflow or aspects of the device that did not function effectively. Responses to the above interview questions were collated and are reported below.

## RESULTS

Six health care workers reported back that the device was perceived as user friendly for first-time users and appreciated the intuitive software interface. Additionally, the benefit of having dedicated user and troubleshooting guides was well appreciated.

A total of 200 adults ages 18 years old and older in the study area were screened, and 187 participants agreed to participate. The device was well accepted by both health care workers and participants. The full procedure from blood collection to result generation was implemented under the observation of the trainers in remote regions of central Gabon during daytime household visits. Procedures were first explained to the participants and community representatives before starting the procedures. Participants and health care workers are used to the procedure of finger pricking as this is performed in standard malaria diagnostics in the region several times per year. The filling of the capillary was at times challenging. Blood viscosity and difficulties drawing sufficient blood into the capillary after a finger prick (particularly when using standard lancets) occasionally resulted in insufficient sampling, resulting in artefacts visualized under microscopy because of air drops in the capillaries. Field-workers did not report any issues with the insertion of the capillary into the NTDscope. Immediately after capillary insertion, videos recording the blood specimen were obtained, and the motion of *L. loa* microfilaria was captured if present. The participant samples included specimens infected with *M*. *perstans*. For the detection of *M. perstans* microfilariae, the NTDscope demonstrated a sensitivity of 87% and a specificity of 85% after excluding cases that were classified as false negatives by thick smear microscopy.^16^

Challenges reported by health care workers included environmental conditions, such as overheating of the device when used during the hottest midday hours, necessitating pauses as one device could not function, and the outdoor use of the device in sunlight, leading to occasional impacted visibility and sample handling. The overheating was communicated to the engineers who discovered that a software problem (which also drained the battery) had to be fixed, and subsequent field tests in Cameroon confirmed that overheating is no longer an issue.

Additionally, the small charger capacity did not allow for the simultaneous operation and charging of the device, and devices had about 4 to 5 hours of battery power when used in field conditions.

Moreover, participants’ feedback indicated that training modules should be made available online for quick and easy reference and so that the training does not depend on trainers from abroad. Additionally, the user and troubleshooting guides were available in English and could be made available in Portuguese, Spanish, and particularly, French as most of the *L. loa*-endemic countries are francophone. Overall, the screening process was completed without significant technical issues during field preparation in a short time. In terms of logistics and availability, access to the device in remote locations proved feasible as the device was light and easy to transport. Moreover, the NTDscope functioned without continuous internet connectivity and was compatible with battery or solar power, which supported the aim of using the device in zero-infrastructure environments.

The Android-based application functioned reliably during the study. Data were first stored locally on the device and subsequently uploaded to the cloud storage system during the night through the institutional Wi-Fi connection or the local mobile phone network connectivity. This process of uploading data was judged to be convenient by the study personnel. During the implementation study, no major confidentiality risks concerning participants’ data and data protection were observed.

The software was able to perform automated analysis rapidly, and no significant delays in generating results were reported. However, a challenge was that the device did not automatically recognize duplicate samples, meaning that sample testing was already performed for the same participant. In general, the NTDscope was well received by field-workers and participants, proving to be an efficient and robust tool under field conditions. The screening process was rapid, with an average of 30 samples screened within an hour. Logistical challenges were manageable, and the system operated effectively without external infrastructure. In contrast, conventional light microscopy would have required multiple steps, including slide preparation, Giemsa, and drying, which alone would have taken 30 to 45 minutes per sample before microscopic examination. The reading process is operator dependent and time consuming, particularly in low-density infections, and it is further limited by the availability of trained microscopists. As a result, the time from blood collection to diagnostic decision may exceed 1 hour per patient. In field settings lacking laboratory infrastructure, this delays treatment initiation and poses challenges for the feasibility of large-scale treatment.

## DISCUSSION

The overall aim of the NTDscope initiative is to develop a cost-efficient, innovative, and rapid point-of-care diagnostic tool that enables both the identification of high-risk participants and geomapping of disease, ultimately facilitating ivermectin large-scale treatment. In this study, we used the latest technical version of the NTDscope in field settings, which did not require sample preparation or a trained microscopist, although results were not available at the point-of-sample preparation in this study. Only a lancet and a capillary per participant were necessary, making the device highly cost efficient in field implementation, with each sample costing less than one U.S. dollar. Moreover, minimal training of health care personnel was required to ensure correct sample handling. Sample collection, preparation, and processing were relatively short, with turnaround times varying between 3 and 5 minutes, enabling rapid and efficient screening.^2^

This implementation study illustrates that health care workers can rapidly be trained to use the NTDscope for *L. loa* screening and can efficiently carry out high-volume testing with high sensitivity and specificity in real-world rural settings in Gabon.^16^ The ability to process multiple patient samples consecutively on a single device further enhances throughput and operational efficiency. This may facilitate public health screening programs in Central Africa and large-scale disease mapping programs in the region.^15^ The teams were able to upload field data into a secure, cloud-based system, and such an approach enables long-term data storage that may be used to guide further public health interventions. Key areas for improvement include enhancing battery capacity to support simultaneous operation and charging as well as optimizing heat dissipation during prolonged use. Modifications to address these issues are already in progress.

The strength of this implementation study was that the NTDscope was for the first time transferred from the laboratory into a real-world, remote setting in Gabon, where it was used by individuals without any experience in either traditional light microscopy or smartphone-based microscopy. This allowed for an authentic assessment of the device’s usability under operational conditions directly informed by end-user feedback.

In a previous study by D’Ambrosio et al.,^15^ which tested the second-generation LoaScope in the region surrounding Yaoundé, Cameroon, 33 potentially *L. loa*-infected patients were tested. D’Ambrosio et al.^15^ confirmed that automated counts correlated with manual thick smear counts, and they reported 94% specificity and 100% sensitivity. To test whether the second-generation LoaScope enabled the safe exclusion of patients from ivermectin-based large-scale treatment at the point of care in *L. loa*-endemic regions, Kamgno et al.^13^ conducted a large-scale study involving 16,259 participants from August to October 2015 in the Okola health district in Cameroon where ivermectin distribution had been suspended since 1999 because of fatal *L. loa*-associated adverse events. In this setting, a test-and-not-treat strategy was implemented using the second-generation LoaScope to identify and exclude high-risk individuals from large-scale treatment with ivermectin.^13^ Evaluation of the NTDscope prototype showed similar results. It demonstrated high diagnostic performance, with sensitivity and specificity comparable with conventional microscopy. Notably, the NTDscope detected several very low-intensity *L. loa* and *M. perstans* infections that were initially classified as negative by microscopists examining thick blood smears. When these microscopy false negatives were reclassified as true positives based on NTDscope detection, the adjusted specificity of the NTDscope increased to 99.2%. This suggests that the NTDscope may overcome certain limitations of the current gold standard, particularly in detecting low-intensity infections.^16^

This implementation study focused on the usability and practical deployment of the NTDscope used for *L. loa* screening in field conditions. As the device remains in its final stages of development, the next step might be to implement the device in the frame of a potentially large-scale treatment in affected countries. It would also be interesting to apply different functions of the NTDscope as the affected countries, like, for example, Gabon, mostly harbor multiple neglected tropical diseases, such as schistosomiasis and soil-transmitted helminths. Future studies will be needed to systematically address these aspects of the NTDscope. It will also be of interest to further evaluate its usefulness for individual patient management and its diagnostic value in detecting other filarial infections.

## CONCLUSION

The NTDscope may be a powerful tool for the future implementation of large-scale treatment as it enables rapid, point-of-care quantification of *L. loa* microfilariae and may significantly facilitate large-scale treatment programs for the elimination of onchocerciasis and lymphatic filariasis in *L. loa*-endemic areas across Central and West Africa. This aligns with the principle of leaving no one behind by enabling safe participation in large-scale treatment strategies, even in the most underserved *L. loa*-endemic regions.
